# Supplementary material for: Robust and interpretable prediction of gene markers and cell types from spatial transcriptomics data
Source: Nat Commun. 2026 Jan 16;17:1781. doi: 10.1038/s41467-026-68487-0 (PMC12917206; doi:10.1038/s41467-026-68487-0)
Supplement: Supplementary file 3 — Reporting Summary [file 41467_2026_68487_MOESM3_ESM.pdf]

nature portfolio

Corresponding author(s): Quan Nguyen

Last updated by author(s): 13/11/2025

Reporting Summary

Nature Portfolio wishes to improve the reproducibility of the work that we publish. This form provides structure for consistency and transparency in reporting. For further information on Nature Portfolio policies, see our Editorial Policies and the Editorial Policy Checklist.

Statistics

For all statistical analyses, confirm that the following items are present in the figure legend, table legend, main text, or Methods section.

n/a	Confirmed	
☐	☒	The exact sample size (*n*) for each experimental group/condition, given as a discrete number and unit of measurement
☐	☒	A statement on whether measurements were taken from distinct samples or whether the same sample was measured repeatedly
☐	☒	The statistical test(s) used AND whether they are one- or two-sided<br>*Only common tests should be described solely by name; describe more complex techniques in the Methods section.*
☐	☒	A description of all covariates tested
☐	☒	A description of any assumptions or corrections, such as tests of normality and adjustment for multiple comparisons
☐	☒	A full description of the statistical parameters including central tendency (e.g. means) or other basic estimates (e.g. regression coefficient) AND variation (e.g. standard deviation) or associated estimates of uncertainty (e.g. confidence intervals)
☐	☒	For null hypothesis testing, the test statistic (e.g. *F*, *t*, *r*) with confidence intervals, effect sizes, degrees of freedom and *P* value noted<br>*Give P values as exact values whenever suitable.*
☒	☐	For Bayesian analysis, information on the choice of priors and Markov chain Monte Carlo settings
☐	☒	For hierarchical and complex designs, identification of the appropriate level for tests and full reporting of outcomes
☐	☒	Estimates of effect sizes (e.g. Cohen's *d*, Pearson's *r*), indicating how they were calculated

Our web collection on statistics for biologists contains articles on many of the points above.

Software and code

Policy information about availability of computer code

Data collection	bcl2fastq version 2.17 and SpaceRanger version 1.0 were used for mapping 10x Visium spatial transcriptomics FASTQ reads to the human reference genome (version GRCh38-3.0.0) to generate the gene expression count matrix for the human skin (FFPE, n=13) samples.<br><br>For the skin cancer Phenocycler Fusion dataset, the imaging protocol was established with the Fusion Experiment Designer software V1.0 according to the manufacturer’s instructions. Following the acquisition of all cycles, a final composite QPTIFF image file was exported from Phenocycler Fusion software V1.0.<br><br>FFFPE breast cancer samples were sectioned at 5 um thickness and mounted on Xenium slides (10x Genomics). In situ gene expression profiling was performed using the Xenium In Situ Platform (10x Genomics) according to the manufacturer's protocol with the Pre-designed Xenium gene panel targeting 280 genes. In brief, tissue sections underwent deparaffinisation and decrosslinking, followed by probe hybridization overnight. The correct-binding probes were circularised by ligation and amplified by rolling cycle amplification. Autofluorescent quench and nuclei staining were performed prior to Xenium Instrument, software version 1.4.2.0. Raw fluorescence images were processed using Xenium onboard Analysis version 1.4.0.6 with default parameters for cell segmentation, transcript decoding, and assignment.<br><br>450 H&E images from 213 patients, randomly selected from TCGA (https://www.cancer.gov/ccg/research/genome-sequencing/tcga).

Data analysis

The data were analysed using our custom algorithm, STimage, which is publicly available on GitHub (https://github.com/BiomedicalMachineLearning/STimage).

For manuscripts utilizing custom algorithms or software that are central to the research but not yet described in published literature, software must be made available to editors and reviewers. We strongly encourage code deposition in a community repository (e.g. GitHub). See the Nature Portfolio guidelines for submitting code & software for further information.

Data

Policy information about availability of data

All manuscripts must include a data availability statement. This statement should provide the following information, where applicable:

- Accession codes, unique identifiers, or web links for publicly available datasets
- A description of any restrictions on data availability
- For clinical datasets or third party data, please ensure that the statement adheres to our policy

The datasets that support the findings of this study are available from several sources. Publicly available datasets used include Her2ST [https://github.com/almaan/her2st], Swarbrick's Lab [https://zenodo.org/records/4739739], Public breast cancer 10X-Visium [https://www.10xgenomics.com/datasets], Liver Visium (GSE240429) [https://www.ncbi.nlm.nih.gov/geo/query/acc.cgi?acc=GSE240429], and Kidney cancer Visium (E-MTAB-12767) [https://www.ebi.ac.uk/biostudies/ArrayExpress/studies/E-MTAB-12767]. A collected and processed compilation of these public datasets is accessible via UQ eSpace [https://doi.org/10.48610/4fb74a9]. Furthermore, TCGA H&E images (TCGA-BRCA) used for model inference are available from the GDC Data Portal [https://portal.gdc.cancer.gov/projects/TCGA-BRCA]. The in-house datasets generated for this study have been deposited in the UQ eSpace repository and are available upon request at [https://doi.org/10.48610/e8426d2]. These include: (1) breast cancer Xenium (raw data and H&E image); (2) melanoma Visium (raw sequencing and processed data); and (3) melanoma PhenoCycler Fusion (raw images and processed data). The raw sequencing data of melanoma Visium data have been deposited in the The European Genome-phenome Archive (EGA) and is accessible under the accession number EGAD50000002172.

Access to these in-house datasets is restricted due to ethical considerations and patient consent limitations. Researchers may request access via the UQ eSpace repository. Access will be granted to researchers who agree to the data use and ethics terms. Requests are typically reviewed within 24 hours, and once approved, access will be provided for a period of 12 months.

Research involving human participants, their data, or biological material

Policy information about studies with human participants or human data. See also policy information about sex, gender (identity/presentation), and sexual orientation and race, ethnicity and racism.

Reporting on sex and gender

The Skin Visium dataset (13 tissue from 10 patients) includes 6 males and 4 females, with donor ages ranging from 59 to 78 years. The Melanoma PhenoCycler Fusion dataset (n=3) consists of 1 male and 2 females, with ages ranging from 53 to 70 years. The breast cancer Xenium dataset includes 5 samples, all of which are from females within the 18–40 age range. Sex and gender were not used in the analysis. Sex was determined based on data assigned in participants' electronic medical records.

Reporting on race, ethnicity, or other socially relevant groupings

No data on race, ethnicity, or other socially relevant groupings were collected for the donors associated with the datasets used in this study. These variables were not used as parameters in any analysis, nor were they controlled for as confounding variables.

Population characteristics

The population characteristics available for the human research participants included age and gender, which are detailed in the sample information tables (e.g., Supplementary Tables [1-3]).

However, these participant-level demographic variables were not used as covariates in our analysis. Our model operates and performs predictions at the tissue level, based on the spatial and molecular features within the tissue samples, and does not incorporate donor population characteristics.

Recruitment

This study did not involve direct participant recruitment. We utilized de-identified human tissue samples from previously established cohort. As participants were not recruited for this specific computational project, self-selection bias is not a relevant factor. While the original cohorts from which these samples were sourced may have inherent selection biases (e.g., collection from specific clinical centers or tissue banks), this is unlikely to impact the technical evaluation and results of our model, which operates at the tissue-feature level.

Ethics oversight

The data generation complied with all relevant ethical regulations and was approved by the University of Queensland’s Human Research Ethics Committees (ethics approval numbers 2018000165 and 2017000318) and by the Metro South Human Research Ethics Committee (11QPAH477) and the The Royal Women's Hospital Human Research Ethics Committee (2005/HE000785).

Note that full information on the approval of the study protocol must also be provided in the manuscript.

Field-specific reporting

Please select the one below that is the best fit for your research. If you are not sure, read the appropriate sections before making your selection.

☒ Life sciences ☐ Behavioural & social sciences ☐ Ecological, evolutionary & environmental sciences

For a reference copy of the document with all sections, see nature.com/documents/nr-reporting-summary-flat.pdf

Life sciences study design

All studies must disclose on these points even when the disclosure is negative.

Sample size	The sample sizes for all experiments performed in this study were determined as follows. For previously published datasets, the sample sizes were dictated by the original study design and were used as provided. For all in-house generated datasets, we aimed to include a minimum of three samples for each dataset.<br>1. HER2+ breast cancer datasets consist of 36 samples from frozen tissues, at 100 µm resolution. It starts with 13,653 unfiltered spots, reduces to 12,584 remaining spots after quality control, and involves 11,871 genes.<br>2. Wu et al. dataset consist 6 samples from frozen tissues at 55 µm resolution, this dataset has 15,611 unfiltered spots, 14,951 remaining spots after quality control, and includes 14,664 genes.<br>3. Public breast cancer 10x Visium (2 samples) includes frozen tissues with a resolution of 55 µm. It includes 7,785 unfiltered spots, of which 7,289 remain after quality control, and captures 36,601 genes.<br>4. Public breast cancer 10x Visium (1 sample) FFPE tissue dataset with 55 µm resolution has 2,218 unfiltered spots, reduces to 2,338 remaining spots after quality control, and 36,601 genes.<br>5. In-house Skin cancer 13 FFPE tissue Visium samples at 55 µm resolution include 15,209 unfiltered spots and 14,405 remaining spots after quality control, involving 17,943 genes.<br>6. Liver Visium 4 samples from frozen tissues at 55 µm resolution start with 19,968 unfiltered spots, reduce to 19,967 remaining spots after quality control, and involve 36,601 genes.<br>7. Kidney cancer Visium 6 frozen tissue samples, measured at 55 µm, include 16,131 unfiltered spots and 15,632 remaining spots after quality control, involving 36,601 genes.<br>8. In-house Breast cancer Xenium 5 samples from frozen tissues measured at subcellular resolution include 1,294,600 cells and 280 genes.<br>9. In-house Skin cancer CODEX 3 sample from frozen tissue at subcellular resolution includes 813,004 cells and captures 33 proteins.<br><br>A formal sample size calculation was not performed prior to these experiments. Instead, our sample size of n≥3 was chosen for two primary reasons. First, this number aligns with the current standards in the rapidly evolving field of spatial biology, where the significant financial cost and technical complexity of data generation often constrain the number of samples. Second, the nature of spatial data itself provides a high-dimensional readout for each sample, with each replicate containing thousands of unique data points (i.e., spots or cells). This inherent data richness within each sample provides sufficient statistical power to support the conclusions drawn from our model.
Data exclusions	No data were excluded from the analyses.
Replication	We use leave-one-out cross-validation to support our findings.
Randomization	Our randomisation strategy involves random shuffling of the spots for training the model, random initialise the model weights for model uncertainty estimation, and random testing on external tissue HE images from the TCGA dataset.
Blinding	All analyses included in this study were data-driven and unsupervised. The functional gene list for training the model was selected from external sources. The model was trained using leave-one-out cross-validation to ensure that the test data were unseen during the model training.

Reporting for specific materials, systems and methods

We require information from authors about some types of materials, experimental systems and methods used in many studies. Here, indicate whether each material, system or method listed is relevant to your study. If you are not sure if a list item applies to your research, read the appropriate section before selecting a response.

Materials & experimental systems

n/a	Involved in the study	
☒	☐	Antibodies
☒	☐	Eukaryotic cell lines
☒	☐	Palaeontology and archaeology
☒	☐	Animals and other organisms
☒	☐	Clinical data
☒	☐	Dual use research of concern
☒	☐	Plants

Methods

n/a	Involved in the study	
☒	☐	ChIP-seq
☒	☐	Flow cytometry
☒	☐	MRI-based neuroimaging

Plants

Seed stocks	*Report on the source of all seed stocks or other plant material used. If applicable, state the seed stock centre and catalogue number. If plant specimens were collected from the field, describe the collection location, date and sampling procedures.*
Novel plant genotypes	*Describe the methods by which all novel plant genotypes were produced. This includes those generated by transgenic approaches, gene editing, chemical/radiation-based mutagenesis and hybridization. For transgenic lines, describe the transformation method, the number of independent lines analyzed and the generation upon which experiments were performed. For gene-edited lines, describe the editor used, the endogenous sequence targeted for editing, the targeting guide RNA sequence (if applicable) and how the editor was applied.*
Authentication	*Describe any authentication procedures for each seed stock used or novel genotype generated. Describe any experiments used to assess the effect of a mutation and, where applicable, how potential secondary effects (e.g. second site T-DNA insertions, mosiacism, off-target gene editing) were examined.*
